# Genetic disorders of thyroid development, hormone biosynthesis and signalling

**DOI:** 10.1111/cen.14817

**Published:** 2022-09-05

**Authors:** Carla Moran, Nadia Schoenmakers, W. Edward Visser, Erik Schoenmakers, Maura Agostini, Krishna Chatterjee

**Affiliations:** ^1^ Wellcome Trust‐MRC Institute of Metabolic Science University of Cambridge Cambridge UK; ^2^ Department of Internal Medicine Erasmus Medical Center, Academic Center for Thyroid Diseases Rotterdam The Netherlands; ^3^ Present address: Beacon Hospital and School of Medicine University College Dublin Ireland

**Keywords:** congenital hypothyroidism, thyroid dysgenesis, thyroid hormone receptors, thyroid hormone resistance, thyroid hormones

## Abstract

Development and differentiation of the thyroid gland is directed by expression of specific transcription factors in the thyroid follicular cell which mediates hormone biosynthesis. Membrane transporters are rate‐limiting for cellular entry of thyroid hormones (TH) (T4 and T3) into some tissues, with selenocysteine‐containing, deiodinase enzymes (DIO1 and DIO2) converting T4 to the biologically active hormone T3. TH regulate expression of target genes via hormone‐inducible nuclear receptors (TRα and TRβ) to exert their physiological effects. Primary congenital hypothyroidism (CH) due to thyroid dysgenesis may be mediated by defects in thyroid transcription factors or impaired thyroid stimulating hormone receptor function. Dyshormonogenic CH is usually due to mutations in genes mediating thyroidal iodide transport, organification or iodotyrosine synthesis and recycling. Disorders of TH signalling encompass conditions due to defects in membrane TH transporters, impaired hormone metabolism due to deficiency of deiodinases and syndromes of Resistance to thyroid hormone due to pathogenic variants in either TRα or TRβ. Here, we review the genetic basis, pathogenesis and clinical features of congenital, dysgenetic or dyshormonogenic hypothyroidism and disorders of TH transport, metabolism and action.

## 1 DISORDERS OF THYROID HORMONE (TH) DEVELOPMENT AND BIOSYNTHESIS

### 1.1 Background

Primary congenital hypothyroidism (CH) is traditionally subdivided into thyroid dysgenesis (TD), failure of normal thyroid development due to thyroid ectopy, athyreosis or hypoplasia and dyshormonogenesis (DH), inadequate TH biosynthesis despite a normally‐sited, often goitrous thyroid. Monogenic causes of TD are rare, occurring in <5% affected cases whereas DH is usually attributable to pathogenic variants affecting known components of the TH biosynthesis pathway.^1^

### 1.2 Thyroid dysgenesis

Monogenic causes of TD predominantly involve pathogenic variants in key thyroidal transcription factors which define developing thyroid follicular cells (*NKX2‐1*, *PAX8* and *FOXE1*), as well as *GLIS3*, and the thyroid stimulating hormone (TSH) receptor (*TSHR*). Since transcription factor expression is not confined to the thyroid, pathogenic variants may cause characteristic, multisystem defects reflecting their extrathyroidal expression whereas pathogenic variants in *TSHR* cause isolated hypothyroidism.^1^

***NKX2‐1***: Monoallelic, pathogenic variants in *NKX2‐1* represent the most common CH‐associated transcription factor defect and cause a variably penetrant ‘brain‐lung‐thyroid’ syndrome for which 50% affected cases exhibit the complete triad. Overall ~70% cases with pathogenic NKX2‐1 variants exhibit hypothyroidism. ~90% exbibit neurological features (typically a benign hereditary chorea), and ~50% have pulmonary involvement (including infant respiratory distress syndrome) which carries a 16% mortality. Although affected individuals may have TD, CH is typically mild with a normal sized, normally‐located gland‐in‐situ (GIS CH). Pathogenic variants frequently occur de novo, and deletions proximal to *NKX2‐1* may also cause brain‐lung‐thyroid syndrome.^2^

***PAX8***: Monoallelic, pathogenic PAX8 variants classically cause thyroid hypoplasia, however, almost 30% affected cases have GIS CH, and a minority exhibit thyroid ectopy or athyreosis. Although associated hypothyroidism is usually congenital, it may also be transient or subclinical or develop after the neonatal period. PAX8 is also expressed in the nephrogenic mesenchyme, and a spectrum of associated urogenital tract abnormalities have been reported in a small minority of cases.^1^, ^3^

***FOXE1***: Pathogenic *FOXE1* variants cause recessively‐inherited CH and the extrathyroidal expression of FOXE1 in oropharynx, oesophagus, choanae and hair follicles underpins a highly penetrant triad of associated developmental abnormalities. Affected individuals typically exhibit athyreosis or severe thyroid hypoplasia, cleft palate and spiky hair and more rarely, choanal atresia or bifid epiglottis. Pathogenic variants are rare and usually impair FOXE1 DNA binding and transcriptional activity but a clinically indistinguishable gain‐of‐function mutant (Arg73Ser), has also been reported.^1^, ^4^

***GLIS3***: Biallelic, pathogenic variants in *GLIS3* are a rare cause of CH associated with a multisystem phenotype consistently including permanent neonatal diabetes. Additional, variably penetrant defects include renal cystic dysplasia, congenital glaucoma, hepatic cholestasis, liver fibrosis and facial dysmorphisms reflecting pleiotropic extrathyroidal roles for GLIS3. Thyroid morphology ranges from apparently normal to athyreosis and in some cases, TSH and TG levels remain elevated during levothyroxine treatment despite normalisation of free T4. Studies in murine and zebrafish models have suggested possible roles for GLIS3 in TSHR signalling and specification, respectively.^5^, ^6^

***TSHR***: TSHR is a G protein coupled receptor which stimulates thyrocyte proliferation and thyroid hormonogenesis. Mono‐ or biallelic inactivating, pathogenic variants in *TSHR* result in a spectrum of TSH resistance which, if complete (e.g., due to biallelic and nonfunctional *TSHR* alleles) results in severe gland hypoplasia and profound CH. Conversely, partial TSH resistance (e.g., due to monoallelic and hypomorphic *TSHR* alleles), results in GIS CH with isolated hyperthyrotropinaemia.

Deleterious, pathogenic variants in *TSHR* occur moderately frequently, with founder mutations reported in certain populations. In individuals harbouring heterozygous mutants causing partial resistance, hyperthyrotropinaemia may compensate for the TSHR defect, and maintain euthyroidism, obviating the need for levothyroxine replacement in some individuals.^7^, ^8^

### 1.3 Additional genes associated with TD

Monoallelic and biallelic pathogenic variants in *CDCA8* and *TUBB1* have recently been implicated in the pathogenesis of TD, and JAG1 may also contribute, especially to orthotopic gland hypoplasia.^9^

### 1.4 Dyshormonogenesis

**Figure 1:**
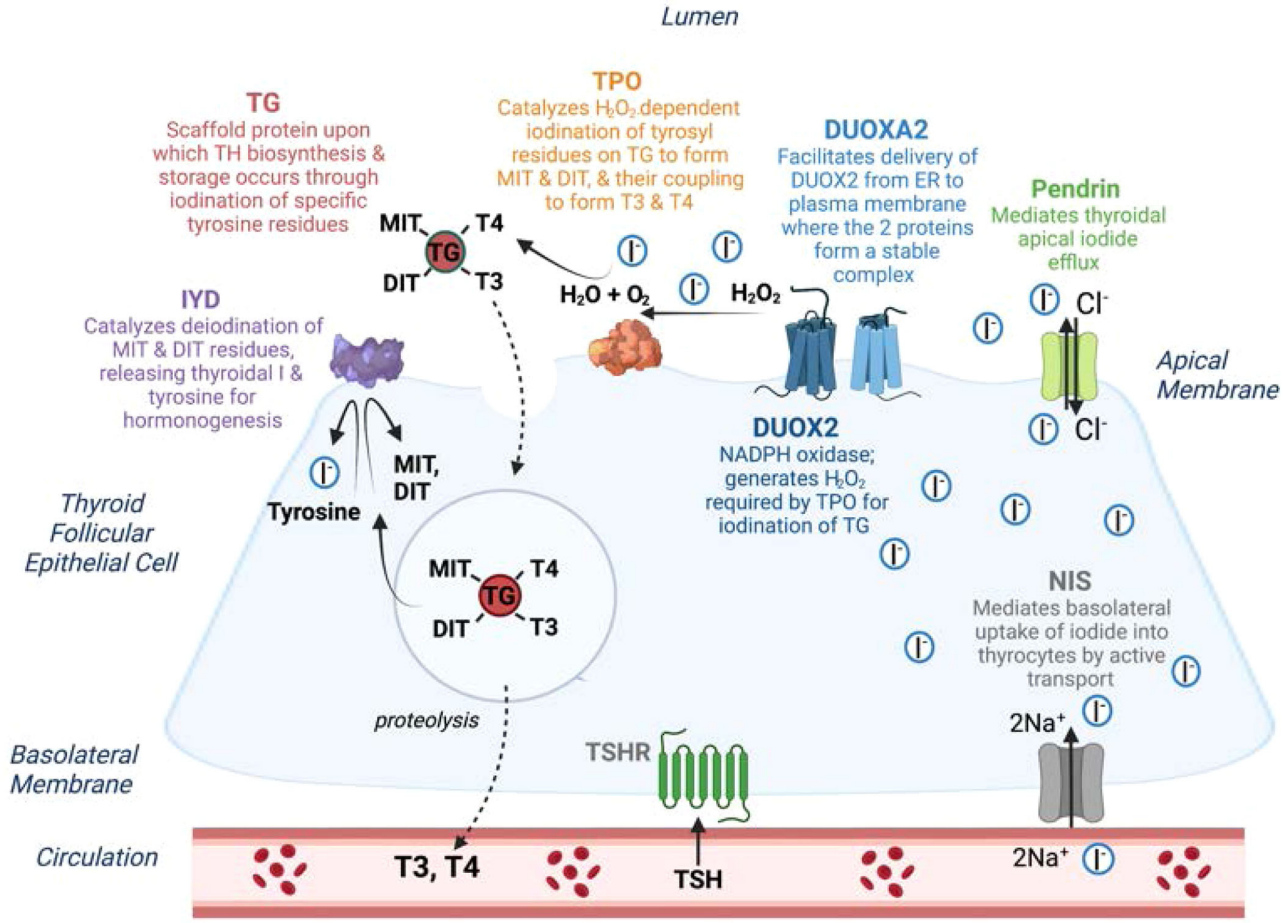
Schematic depicting a thyroid follicular cell and the process of thyroid hormone (TH) biosynthesis: Circulating iodide (I^−^) is transported across the basolateral membrane by the sodium‐iodide symporter (NIS, SLC5A5), and I^−^efflux across the apical membrane is mediated by specific transporters including Pendrin. In the follicular lumen, I^−^ is oxidized in the presence of hydrogen peroxide (H_2_O_2_), generated by DUOX2 (an NADPH‐oxidase enzyme) and its accessory protein, DUOXA2. TPO catalyses the oxidation of I^−^ into I^+^, the iodination of tyrosyl residues on the surface of TG to form mono and di‐iodotyrosyl (MIT and DIT) and the coupling of MIT and DIT to produce TH (thyroxine (T4) and triiodothyronine (T3). TG‐bound T3 and T4 are endocytosed back into the thyroid follicular cell then cleaved and secreted into the circulation; iodotyrosine deiodinase (IYD) recycles unused iodide moieties. SLC26A7, an anion transporter, has also been identified as an essential component of the TH biosynthesis machinery, but its molecular role in the thyroid has not yet been determined. Pathogenic variants in any of these proteins can result in dyshormonogenesis. This figure was created in BioRender.com. [Color figure can be viewed at wileyonlinelibrary.com]

TH biosynthesis requires a complex pathway of enzymes and transporter molecules permitting uptake, concentration and organification of circulating iodide, as well as TG substrate for iodination (Figure 1). Pathogenic variants in genes encoding these components (*TG*, *TPO* and *SLC26A4* [*Pendrin*], *SLC5A5* [*NIS*], *DUOX2*, *DUOXA2*, *IYD* and *SLC26A7*) may result in DH, sometimes with associated goitre. Although each genetic defect is associated with key biochemical and radiological hallmarks (Table 1), genetic subtypes of DH are increasingly recognised to show a more variable and broader phenotype than initially appreciated and in many cases it may be difficult to predict the genetic defect from these clinical features.^1^, ^10^, ^11^, ^12^

**Table 1:** Genetic causes of dyshormonogenic hypothyroidism

Gene	Inheritance, Epidemiology	Hallmarks of associated CH
TG	Biallelic Frequent cause of DH	Biochemistry: severe CH to euthyroidism; frequent goitre Inappropriately low serum TG despite ↑TSH/failure of exogenous TSH to stimulate TG rise. T3 levels may be paradoxically normal/mildly ↑, with ↓/low‐normal T4, & ↑T3/T4 ratio Thyroidal I^‐^uptake:^a^ I^‐^organification: usually preserved.
TPO	Biallelic^b^ Frequent: commonest cause of TIOD^c^	Biochemistry: often severe CH; frequent goitre Thyroidal I^‐^uptake:^a^ I^‐^organification:↓ (usually TIOD).^c^
SLC5A5 (NIS)	Biallelic Rare cause of DH	Biochemistry: severe CH to euthyroidism; frequent goitre Decreased saliva:plasma iodine ratio May be delayed TSH rise^d^ Thyroidal I^‐^uptake:↓
DUOX2	Mono‐/biallelic Frequent cause of DH, especially in East Asians. Mutant allele frequency ~ 1% in certain populations	Biochemistry: transient^e^/mild permanent CH (Highly variable penetrance and expressivity; biallelic truncating and monoallelic pathogenic variants may both cause transient^e^ and permanent CH.) May be delayed TSH rise^d^ Thyroidal I^‐^uptake:^a^ I^‐^organification: ↓ (usually PIOD)
DUOXA2	Mono‐/biallelic Rare cause of DH	Biochemistry: transient^e^/mild permanent.(clinical data is sparse) Thyroidal I^‐^uptake:^a^ I^‐^organification: ↓ (PIOD)
SLC26A4 (Pendrin)^f^	Biallelic Frequent cause of DH	Biochemistry: Thyroid dysfunction/goitre rare before puberty Only ~50%patients exhibit subclinical or overt hypothyroidism. Thyroidal I^‐^uptake:^a^ I^−^ organification:↓ (usually PIOD)
SLC26A7	Biallelic Rare cause of DH	Biochemistry: Moderate‐severe CH. Frequent Goitre. May be delayed TSH rise^d^ Thyroidal I^−^uptake:^a^ I^−^ organification: ↓ (usually PIOD)
IYD	Mono‐/biallelic Rare cause of DH	Biochemistry severe CH to euthyroidism. Goitre. May be delayed TSH rise^d^ Raised urinary MIT and DIT Thyroidal I^‐^uptake:^a^ (rapid) I^−^organification:usually^a^

Abbreviations: IYD, iodotyrosine deiodinase; PIOD, partial iodide organification defect; TSH, thyroid stimulating hormone.

^a^ Preserved;

^b^ Heterozygous, pathogenic variants in TPO are rarely associated with milder hypothyroidism and have rarely been reported in association with TIOD, possibly due to monoallelic expression of mutant TPO in thyroid;

^c^ TIOD, total iodide organification defect (release of >90% accumulated intrathyroidal radiodine during a perchlorate discharge test);

^d^ Delayed TSH rise, Newborn screening TSH levels may be normal followed by delayed development of biochemical hypothyroidism;

^e^ Transient CH, CH diagnosed at birth which spontaneously remits as thyroid hormone biosynthesis requirements decrease in early childhood, permitting cessation of levothyroxine treatment;

^f^ Pathogenic variants in Pendrin also cause congenital sensorineural hearing impairment with enlargement of the vestibular aqueduct (pendred syndrome when associated with goitre and PIOD).

### 1.5 Diagnosis, molecular genetics and clinical management

Untreated CH results in profound neurodevelopmental delay therefore most industrialized countries operate neonatal screening programmes for CH, diagnosing the majority of affected individuals shortly after birth on the basis of an elevated TSH level and free T4 concentration below the age appropriate reference range. The mainstay of therapy in CH is levothyroxine, which should be initiated promptly following diagnosis, and adjusted frequently during childhood to maintain biochemical euthyroidism. Making a genetic diagnosis can clarify recurrence risk and inform reproductive options for disorders with irreversible, detrimental consequences.

Genetic evaluation is most likely to yield a molecular diagnosis in CH when DH is suspected or where clinical features support a *TSHR* or transcription factor defect. In these settings, genetic ascertainment can inform appropriate counselling for disorders where multisystem involvement is anticipated (e.g., *NKX2‐1, FOXE1, GLIS3*, and *Pendrin* mutations), or permit tailored treatment, with withdrawal of levothyroxine in childhood if CH is likely to be transient (e.g., *DUOX2/DUOXA2*‐mediated CH) or in individuals with hyperthyrotropinaemia due to heterozygous *TSHR* mutations who may not require treatment at all.^1^, ^8^, ^10^ Furthermore, establishing a genetic aetiology in CH with a delayed TSH rise, may enable prompt diagnosis in affected siblings to prevent neurodevelopmental delay.

Although CH may have a monogenic basis, molecular diagnosis is optimised by the use of next generation sequencing (NGS) technologies which permit a nonhypothesis‐driven approach, thus overcoming the difficulty of predicting genetic aetiology on clinical grounds alone. Additionally, NGS permits the identification of oligogenic causes for CH, which have recently been shown to play a major role in the pathogenesis of both TD and DH. Oligogenic inheritance may also explain in part both the apparently sporadic occurrence of TD, and the frequent variable expressivity and penetrance of causal mutations in CH.^9^, ^13^

## 2 DISORDERS OF THYROID HORMONE TRANSPORT

### 2.1 MCT8 deficiency

TH transporter proteins at the plasma membrane govern intracellular bioavailability of TH (Figure 3A). Among the transporters identified, only a minority exhibit high specificity towards THs.^14^ Monocarboxylate transporter 8 (MCT8; solute carrier family 16A2, *SLC16A2*, localized at the X‐chromosome) transports T4, T3, rT3 and 3,3'‐T2 and is highly expressed in the brain as well as in the thyroid, liver, kidney and pituitary.^15^ MCT8 deficiency (or Allan‐Herndon‐Dudley syndrome) is a severe disorder with neurological and metabolic sequelae, due to pathogenic variants in MCT8 with an estimated prevalence of 1/70,000 males.^16^, ^17^ Median survival is 35 years, with 30% of patients having died in childhood with pulmonary tract infections, aspiration pneumonia and sudden death being important causes of mortality.^18^

### 2.2 Clinical phenotype

First symptoms typically manifest around 4 months of age. Reasons for referral include developmental delay, hypotonia, poor weight gain and feeding problems. Key clinical features comprise global hypotonia with poor head control as well as upper truncal instability, hypokinesia and dystonic posturing of limbs starting in the first year of life. Both dystonia and spasticity contribute to exaggerated deep tendon reflexes and hypertonia, followed by the development of scoliosis. Early motor milestones (e.g., sitting or walking) are not reached. Patients exhibit moderate‐to‐severe intellectual disability with pronounced delay in speech development. Primitive reflexes (e.g., glabellar reflex) do not disappear over time. Electroencephalogram‐proven seizures are present in approximately a quarter of patients.

Body weight shows deterioration over time with the majority being severely underweight. Cardiovascular dysfunction includes systolic hypertension, tachycardia and frequent premature atrial contractions; conduction abnormalities are also observed more frequently than in the general population.

The endocrine hallmark of MCT8 deficiency is a combination of elevated serum (F)T3 concentrations, low or low‐normal serum (F)T4 concentrations, low rT3 concentrations and normal serum TSH concentrations. In neonatal screening samples, T4 concentrations are low but T3 and TSH concentrations are not elevated, representing potential to identify patients at birth.^18^, ^19^

Brain magnetic resonance imaging (MRI) scanning reveals a global delay in myelination which improves with age. In addition, diffuse atrophy is present with concomitant dilatation of the ventricles.^18^, ^20^

In a minority of patients, the clinical phenotype is less severe. Such patients retain the ability to maintain head control, sit independently, walk (with support) and develop some speech.

### 2.3 Molecular genetics

**Figure 2:**
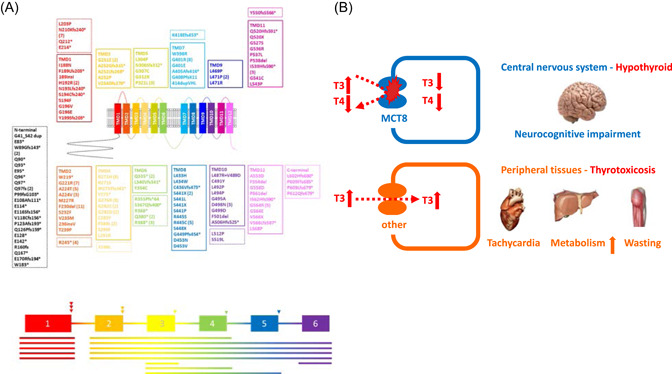
(A) Overview of unique pathogenic variants identified in *SLC16A2* encoding the MCT8 transporter. Different coloured boxes depict the location of different missense, nonsense and frame shift variants in transmembrane domains (TMDs; Solid boxes) or intracellular or extracellular loops (Dashed boxes) of the protein (top of picture). Large deletions (lines) and splice site variants (arrow heads) are superimposed on a schematic of the genomic organisation of *SLC16A2* (bottom of picture). The frequency of pathogenic variants occurring more than once in independent families is in brackets. Three letter amino acid codes which correspond to the single letter codes shown denoting variants, are as follows: A, Ala; C, Cys; D, Asp; E, Glu; F, Phe; G, Gly; H, His; I, Ile; K, Lys; L, Leu; M, Met; N, Asn; P, Pro; Q, Gln; R, Arg; S, Ser; T, Thr; V, Val; W, Trp; Y, Tyr; *, Ter; Δ, Del; fs, Frame shift. (B) Pathophysiology of MCT8 deficiency. MCT8‐dependent cells (brain) are in a hypothyroid state; MCT8‐independent cells (peripheral tissues) are in a thyrotoxic state, being exposed to the increased serum T3 concentrations. The thyroid hormones outside cells reflect the circulating hormone concentrations. [Color figure can be viewed at wileyonlinelibrary.com]

Approximately 150 different pathogenic variants in MCT8 have been reported, with most literature mapping variants onto the long isoform,^14^ which can be classified in four groups: large deletions resulting in an incomplete MCT8 protein, insertions/deletions/nonsense variants resulting in a frame shift or premature truncation, splice site and missense variants resulting in a single amino acid change (Figure 2A). Whereas deletion and truncation variants are obviously pathogenic, this cannot be inferred simply from the nature of splice site or missense variants. Accordingly, assessing the impact of such variants on TH transport requires functional testing using in vitro systems (missense variants) or patient‐derived cells (all variants).

No obvious phenotypic abnormalities have been reported in female carriers, except for FT4 concentrations being intermediate between male patients and noncarriers. Rarely, features of MCT8 deficiency can be present in females resulting from a pathogenic variant in the context of skewed X‐inactivation.

### 2.4 Mechanisms of disease

Depending on the expression of MCT8 and other TH transporters, tissues are either in a hypothyroid state (e.g., brain) or are exposed to toxic T3 concentrations (e.g., liver and muscle) (Figure 2B).

The elevated circulating T3 concentrations contribute to adverse clinical sequelae in tissues (e.g., liver, muscle and heart) where hormone transport is not MCT8 dependent. Based on studies in *Mct8* knockout (KO) mice, different mechanisms that are not mutually exclusive may account for abnormal thyroid function tests: (i) elevated DIO1 activity contributes to high circulating T3 concentrations^21^; (ii) intrathyroidal T4 and T3 concentrations are increased and less T4 is secreted^22^, ^23^; (iii) T4 is trapped in kidneys.^24^ Both the hypothalamus and pituitary are relatively insensitive to TH.^22^, ^23^

With MCT8 being expressed at the blood‐brain barrier, defectiveness of this transporter precludes entry of TH into the brain.^25^ Furthermore, MCT8 is expressed in other cells of the brain (e.g., neurons, astrocytes and tanycytes lining the third ventricle) with cell‐autonomous roles for MCT8.^26^ Therefore, given the critical role of TH in many processes mediating normal brain development, MCT8 deficiency disrupts neurodevelopment.

### 2.5 Clinical management

Supportive care is warranted to address common clinical features (e.g., seizures may require antiepileptic drug therapy; anticholinergic drugs can empirically alleviate dystonia and drooling). Low body weight and swallowing difficulties may require nutritional supplementation (e.g., via percutaneous endoscopic gastrostomy).

Ideally, any treatment should improve or prevent the neurocognitive phenotype and alleviate peripheral thyrotoxicosis. A combination of propylthiouracil (PTU) (but not methimazole) and levothyroxine treatment can improve peripheral thyrotoxicosis, but is not likely to improve brain development. Given the risk hepatic failure, PTU is not recommended as therapy for hyperthyroidism in children.

TH analogues that are not dependent on MCT8 for cellular entry could prevent or reverse the neurological phenotype whilst simultaneously lowering endogenous TH concentrations by inhibiting TSH secretion. Different T3 analogues (Triac [triiodothyroacetic acid], DITPA [diiodothyropropionic acid] and sobetirome and its prodrug Sob‐AM2) have been investigated in (pre)clinical studies with varying effects on different outcomes.^27^, ^28^, ^29^ Substantial clinical experience with Triac therapy of both adults and children has been obtained.^30^, ^31^ Triac treatment lowers elevated T3 concentrations markedly, with consequent, sustained improvements in body weight, heart rate and blood pressure. An ongoing trial (NCT02396459) may determine whether Triac administration in early childhood can modify brain development. The therapeutic potential of other analogues, chaperone drugs or gene therapy remains to be evaluated.

### 2.6 OATP1C1 deficiency

Recently, the first patient with a homozygous, pathogenic variant (Asp252Asn) in the OATP1C1 (*SLCO1C1*) T4‐transporter has been reported.^32^ The clinical phenotype comprised delayed development followed by the progressive loss of acquired skills, ultimately resulting in the absence of speech, spasticity and swallowing difficulties. Cold intolerance was prominent. Serum thyroid function tests were normal. MRI scanning of the brain showed progressive atrophy; an fluorodeoxyglucose‐positron emission tomography scan showed decreased glucose metabolism.

Mechanisms mediating the clinical phenotype are unresolved.^33^, ^34^ The Asp252Asn variant impairs transporter trafficking to the cell membrane, resulting in reduced cellular T4 entry. If the clinical manifestations are attributable to perturbed TH action, it is tempting to speculate that reduced T4 levels in OATP1C1‐expressing astrocytes, resulting in less conversion to T3 by DIO2 present in these cells, leads to insufficient availability of T3 for neighbouring neurons.

A combination of levothyroxine and Triac treatment reportedly improved alertness and swallowing.^32^ Identification of more patients with *OATP1C1* mutations will help further define the clinical phenotype and pathogenetic mechanisms underlying OATP1C1 deficiency.

## 3 DISORDERS OF THYROID HORMONE METABOLISM

### 3.1 Multisystem disorders due to deficiency of selenocysteine (Sec)‐containing proteins

**Figure 3:**
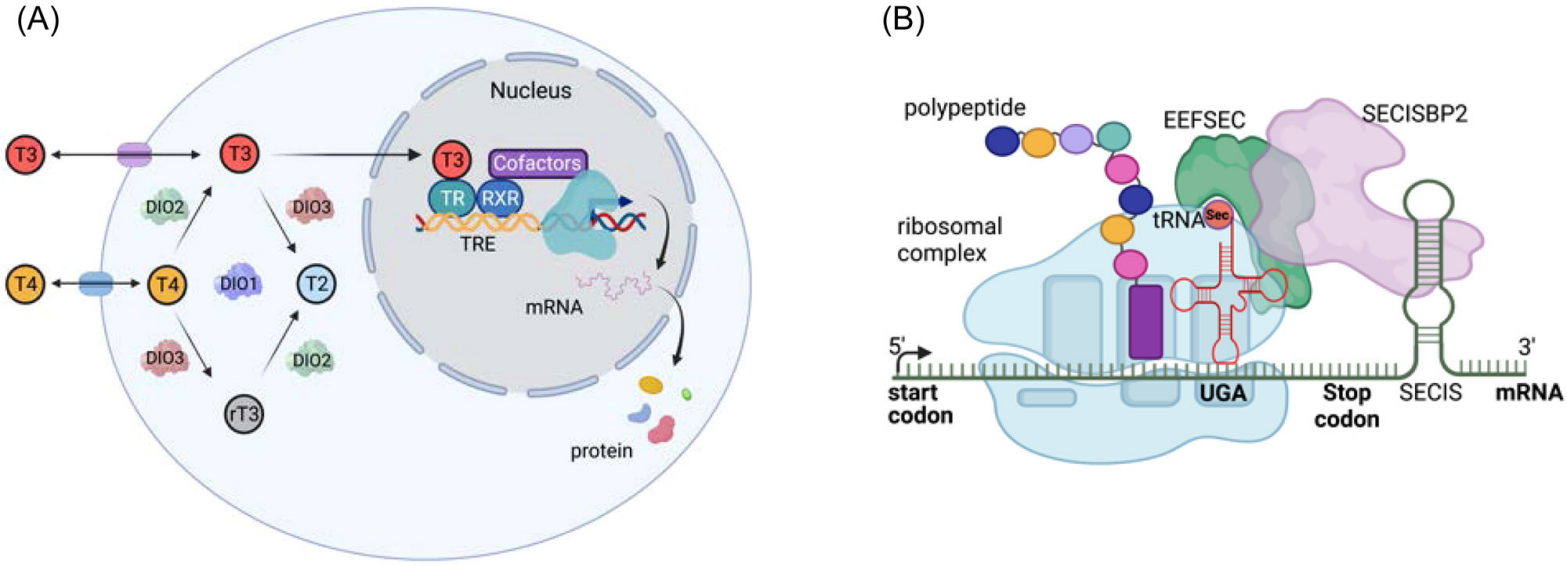
(A) Transport, deiodination and nuclear action of thyroid hormones. Transporters are required for passage of T3 and T4 across the plasma membrane, facilitating hormone uptake, efflux or both. Deiodinase enzymes catalyse conversion of T4 to T3 (DIO1 and DIO2) or inactivation of T4 to rT3 and rT3 to T2 (DIO3). T3 binding to its nuclear receptor (TR), usually part of a heterodimer with RXR, enables recruitment of cofactors which alter transcription of target genes, regulating synthesis of encoded proteins. This figure was created in BioRender.com. (B) Mechanism of selenoprotein biosynthesis. The 3'‐untranslated region of selenoprotein messenger RNAs contains a stem‐loop RNA structure (SECIS element) which interacts with a protein complex that includes SECISBP2 and Sec‐specific elongation factor (eEFSec), enabling a stop codon (UGA) to be recoded, with recruitment of selenocysteyl‐transfer RNA (tRNA^Sec^) to the ribosome and incorporation of selenocysteine (Sec) into the nascent polypeptide. Failure of this mechanism results in the UGA being read as a stop codon, terminating protein synthesis. This figure was created in BioRender.com. SECIS, SElenium Cysteine Insertion Sequence. tRNA, transfer RNA. [Color figure can be viewed at wileyonlinelibrary.com]

Selenium, an essential micronutrient, exerts most of its biological effects as the amino acid Sec, being incorporated into 25 different human selenoproteins and mediating their catalytic enzymatic activity, as oxidoreductases involved in combating either oxidative stress or controlling protein folding pathways in endoplasmic reticulum (ER). The incorporation of Sec into selenoproteins during their translation, involves an unique mechanism in which interaction of SElenium Cysteine Insertion Sequence (SECIS) elements, in the 3'‐UTR of their messenger RNAs with SECIS binding protein 2 (SECISBP2), recodes UGA codons as Sec rather than stop codons, enabling recruitment of transfer RNA (tRNA)^[Ser]Sec^ (encoded by *TRU‐TCA1‐1*) and Sec tRNA‐specific eukaryotic elongation factor (EEFSEC) to the ribosome^35^, ^36^ (Figure 3B).

To date, 18 pathogenic variants in *SECISBP2* (three missense, others frameshift or premature stop) have been recorded in 13 individuals from 11 families from diverse ethnic backgrounds, all exhibiting similar clinical phenotypes.^35^ Consistent with a recessive mode of inheritance, patients are either homozygous (*n* = 3) or compound heterozygous (*n* = 10), with heterozygotes not exhibiting any discernible clinical phenotype. Consistent with known, embryonic lethality of *Secisbp2* KO mice and SECISBP2 being an obligate, limiting, factor for selenoprotein synthesis, cells from patients exhibit reduced selenoprotein expression, probably due to *SECISBP2* hypomorphism, with residual and low‐level, synthesis of functional SECISBP2 protein.

Two, unrelated patients with a homozygous pathogenic variant in *TRU‐TCA1‐1* (Cytosine65Guanine) have been identified,^37^, ^38^ with clinical phenotypes shared with that seen in *SECISBP2* deficient patients. However, patterns of selenoprotein deficiency differ in the two disorders, with relatively preserved synthesis of essential, cellular selenoproteins (e.g., TXNRDs and GPX4) in *TRU‐TCA1‐1* mutant patients, but global selenoprotein deficiency in *SECISBP2* mutant cases.

Most *SECISBP2* cases and one *TRU‐TCA1‐1* patient were diagnosed in childhood with growth retardation and developmental delay. All patients exhibit a characteristic pattern of abnormal thyroid function tests, with raised serum FT4, normal or low FT3, normal or slightly raised TSH and elevated reverse T3 concentrations, reflecting deficiency of all three Sec‐containing deiodinase enzymes (Figure 3A). This pattern of abnormal thyroid function, together with low plasma selenium levels, reflecting decreased levels of the major circulating selenoproteins (SELENOP, GPX3), provides a biochemical signature whereby selenoprotein deficiency due to pathogenic variants in *SECISBP2* or *TRU‐TCA1‐1* can be identified.^35^, ^39^, ^40^

Muscle weakness is another childhood manifestation, contributing to fatigue and motor incoordination. This phenotype, resembling muscular dystrophy due to mutations in selenoprotein N,^41^ affects axial and proximal limb muscles, with elevation of skeletal muscle‐specific creatine kinase (CK‐MM) levels and fatty infiltration of muscle groups (adductor and sartorius), before onset of clinical symptoms.

Azoospermia with spermatogenic maturation arrest, seen in one, adult *SECISBP2* patient, can be attributed to deficiency of several selenoproteins (GPX4, TXNRD3 and SELV), with recognized roles in spermatogenesis.^40^

Bilateral, high‐frequency and sensorineural hearing loss seen in some patients, is progressive with adults being more severely affected. Increased whole body, subcutaneous fat mass and high circulating adiponectin levels are paradoxically associated with enhanced systemic insulin sensitivity, low intrahepatic lipid and possible propensity to spontaneous hypoglycemia in one childhood case.^40^ These phenotypes, together with cutaneous photosensitivity are likely mediated by damage due to raised cellular reactive oxygen species, secondary to deficiencies of Sec‐containing antioxidant enzymes (GPXs and TXNRDs) or selenoproteins protecting against ER stress. The progressive nature of many phenotypes (e.g., hearing loss and muscle weakness), worsening with advancing age, may reflect cumulative oxidative and ER stress‐mediated damage in cells and tissues of patients. Furthermore, it is conceivable that such cumulative damage could also predispose to other phenotypes (e.g., premature ageing and cancer) which have not yet manifested in the relatively young cohort of patients identified hitherto (Table 2).

**Table 2:** Selenoprotein deficiency results in a multisystem disorder with a thyroid signature

Phenotype^a^	Selenoprotein	Function
*Raised FT4, normal/low FT3*	DIO1, DIO2 and DIO3	Thyroid hormone metabolism
*Normal TSH*		
*Raised reverse T3*		
*Low plasma selenium*	SELENOP and GPX3	Plasma selenoproteins
*Muscular dystrophy*	SELENON	Skeletal Muscle
Azoospermia	SELENOV, GPX4 and TRXR3	Spermatogenesis
Photosensitivity	GPXs, TRXRs and MSRB1	Antioxidant enzymes
Increased fat mass and function		
Sensorineural hearing loss		

^a^ Italicised phenotypes have been recorded in both *SECISBP2* and *TRU‐TCA1‐1* defect cases.

In *SECISBP2* cases, treatment with liothyronine can correct subnormal FT3 levels and, alone or in combination with growth hormone, can improve growth and development,^42^, ^43^ although untreated cases ultimately reach normal target height. Administration of the antioxidant alphatocopherol (vitamin E) reduces circulating markers of oxidative damage,^44^ with longer‐term effects yet to be ascertained. Oral selenium supplementation is ineffective in *SECISBP2* cases^45^ but is known to alter the production of Sec‐tRNA^[Ser]Sec^ subtypes,^46^ such that its role in *TRU‐TCA1‐1* defect cases remains to be evaluated.

SEPSECS is essential for Sec‐tRNA^[Ser]Sec^ generation and homozygous or compound heterozygous pathogenic variants cause autosomal recessive pontocerebellar hypoplasia type2D (also known as progressive cerebellocerebral atrophy).^47^ The severity of this neurological phenotype precludes in depth studies, but the published literature suggests that selenoprotein expression is reduced in brain tissue but not other cell types (fibroblasts and muscle cells), with normal circulating T4 and selenium levels in some cases.^35^

### 3.2 Iodothyronine deiodinase type 1 (DIO1) mutations

Pathogenic variants in *DIO1* have been described in two unrelated families.^48^ Raised TSH and positive anti‐TPO antibodies in a proband with Down's syndrome, prompted detailed evaluation of thyroid status in family members. Elevated circulating reverse T3 (rT3) and rT3/T3 ratios (reflecting reduced clearance of rT3 by DIO1) in the asymptomatic proband and family members, cosegregated with heterozygosity for a loss‐of‐function *DIO1* variant (Asn94Lys). Investigation for TSH resistance (without a *TSHR* defect) in another index case, identified a different, loss‐of‐function *DIO1* variant (Met201Ile) in the proband and family members with raised serum rT3, rT3/T3 ratios and total cholesterol levels. In a family with dyshormonogenetic CH due to *TPO* defects, heterozygosity for an additional, deleterious *DIO* variant (Arg132His), correlated with raised circulating T4 relative to T3 and elevated rT3 levels.^49^

## 4 DISORDERS OF THYROID HORMONE ACTION

TH regulate physiological processes (skeletal growth, maturation of the central nervous system, heart rate and contractility and energy expenditure) via receptors (TRα1, TRβ1 and TRβ2) (Figure 3A) encoded by separate genes (*THRA* and *THRB*), with differing tissue distributions: TRα1 is highly expressed in the central nervous system, myocardium, skeletal muscle, bone and gastrointestinal tract; TRβ1 is the predominant receptor subtype in liver and kidney; TRβ2 expression is restricted principally to the hypothalamus, pituitary, retina and inner ear. Such divergence of receptor subtype expression likely mediates distinctive phenotypes associated with defective *THRB* or *THRA*.

### 4.1 Resistance to thyroid Hormone β (RTHβ)

The syndrome that is now known as RTHβ was first described in 1967 when a family with deaf‐mutism, stippled epiphyses, goitre and raised protein bound iodine was reported.^50^ Uniquely, in this family where the disorder is recessively inherited, the molecular basis was shown to be a homozygous deletion encompassing the *THRB* locus. Most commonly, RTHβ is dominantly inherited and over 900 families have been reported, with the population frequency of the disorder estimated to be between 1 in 19,000 and 40,000.^51^, ^52^

### 4.2 Molecular genetics

**Figure 4:**
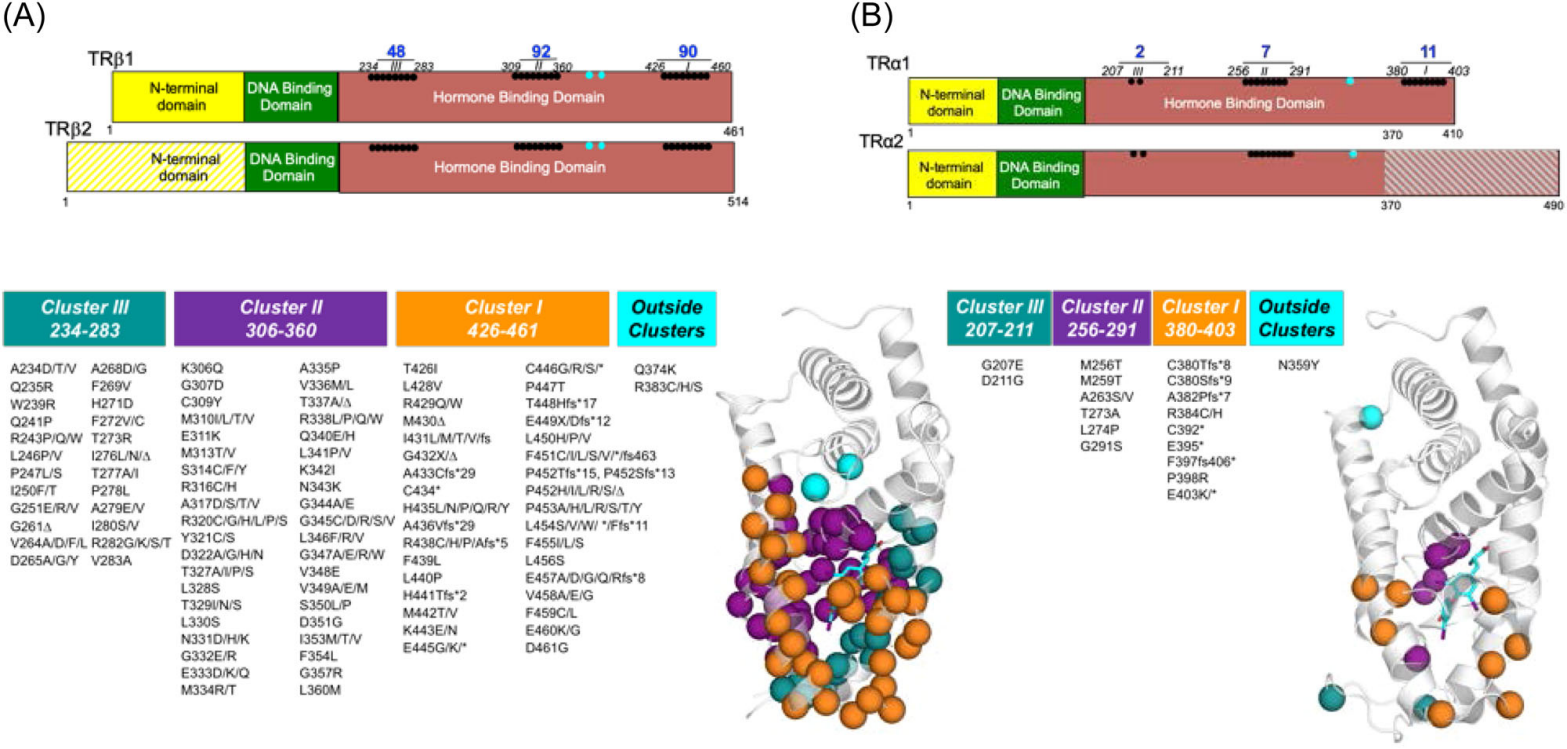
(A) Schematic representation of domains of thyroid hormone β receptor subtypes (TRβ1 and TRβ2), showing that with two exceptions (cyan symbols) all pathogenic variants causing RTHβ described hitherto, localize to three clusters within the hormone binding domain, affecting both TRβ1 and TRβ2 subtypes. The crystal structure of the TRβ hormone binding domain (Protein Data Bank accession no. 1BSX) composed of 12 α‐helices (grey) is shown, with the location of pathogenic variants associated with RTHβ (Cluster I orange, Cluster II purple, Cluster III blue and exceptions cyan) superimposed. As predicted from their functional properties, the majority of deleterious variants involve residues which surround the ligand (T_3_ cyan) binding cavity. (B) Schematic representation of the domains of thyroid hormone receptor alpha 1 (TRα1) and the non hormone binding (TRα2) protein, showing that with one exception (cyan symbol) the smaller number of pathogenic variants causing RTHα identified to date, also localise to three regions within its hormone binding domain, with carboxyterminal variants affecting only TRα1 and other variants being common to both TRα1 and variant α2 proteins. The crystal structure of the hormone binding domain of TRα1 (Protein Data Bank Accession no. 2H79), showing the position of pathogenic variants associated with RTHα, with colour coding denoting that many TRα mutants are equivalent to amino acid changes in TRβ that are known to cause RTHβ and localise within similar clusters. Three letter amino acid codes which correspond to the single letter codes denoting variants shown, are as in the legend to Figure 2A. No RTHα or β receptor mutants, occurring in receptor regions which mediate functions (DNA binding, dimerisation with RXR, corepressor interaction) that are required for dominant negative activity, have been described. RTH, Resistance to thyroid hormone.

Over 230 different heterozygous pathogenic variants in TRβ (mostly missense but also frame shift and premature stop) have been recorded to date,^51^ (Figure 4A). Approximately 10%−15% of patients with clinical and biochemical findings consistent with RTHβ have no identifiable variant in *THRB*; diagnostic possibilities in these individuals include somatic mosaicism for a TRβ variant not expressed in all tissues, a defect in another, unknown gene mediating TH action, or a microscopic, TSH‐secreting, lesion in the pituitary which has yet to manifest radiologically.^51^ *THRB* defects are dominantly‐inherited in most families, but occur sporadically due to *de novo* variants in 10% of cases. All pathogenic *THRB* variants causing RTHβ that have been identified hitherto, cluster within three ‘hotspot’ regions within the hormone binding domain of TRβ, affecting the function of both β1 and β2 receptor subtypes^51^ (Figure 4A). When coexpressed in cells, TRβ mutants inhibit the function of their wild type counterparts in a dominant negative manner. It has been suggested that naturally‐occurring *THRB* variants, localising to other domains of TRβ, may lack such dominant negative activity and therefore be non pathogenic. Very rarely, homozygous, pathogenic TRβ variants, resulting in a more severe clinical and biochemical phenotype, have been described.^53^

### 4.3 Clinical phenotype

The hallmark of RTHβ is refractoriness to action of TH via the β form of the receptor, which is defective. Thus resistance to hormone action within the hypothalamic−pituitary−thyroid axis results in persistent, nonsuppressed synthesis of TSH in the face of elevated, circulating TH; conversely, action of elevated TH via normal TRα, results in hyperthyroidism of TRα‐expressing tissues. Overall, patients exhibit clinical features due to a combination of both insensitivity and overexposure to TH. Many patients are asymptomatic and diagnosed following thyroid function testing for symptoms unrelated to thyroid dysfunction.

In childhood, problems with attention and concentration may occur, as can growth retardation, failure to thrive and goitre.^54^ Both children and adults may experience palpitations, and tachycardia and atrial fibrillation is more common than in healthy individuals,^55^ likely due to cardiac exposure to high TH levels. In severe cases, cardiomyopathy is described.^53^ Middle ear and upper airway tract infections are common.^54^ Hepatic resistance to TH action manifests as normal, circulating sex hormone binding globulin (SHBG) and mixed dyslipidaemia. Systemic insulin resistance and ectopic lipid deposition in tissues (liver and skeletal muscle) has also been described in these individuals.^56^ The prevalence of positive thyroid autoantibodies is higher in RTHβ, suggesting an increased predisposition to thyroid autoimmunity.^57^ Bone mineral density is reduced in adults with RTHβ (Mitchell, Schoenmakers, Moran, Chatterjee unpublished observation). Although cases of (usually microscopic) thyroid cancer in RTHβ patients have been described,^52^ risk of thyroid neoplasia is not overtly increased.

### 4.4 Diagnosis

The biochemical hallmark of RTHβ comprises true (nonartefactual) hyperthyroxinaemia (raised T4 and T3) with non‐suppressed TSH levels (TSH is usually normal or slightly raised). However, this TH pattern can also be caused by other factors such as assay interference (e.g., antiiodothyronine or TSH antibodies, familial dysalbuminaemic hyperthyroxinaemia and displacement of TH from binding proteins) or a TSH‐secreting pituitary tumour. Distinguishing between these entities can be challenging, requiring further studies including biochemical analyses to exclude assay interference, dynamic endocrine investigation (e.g., TRH stimulation and T3 suppression testing) or pituitary imaging.^58^ Following exclusion of assay interference, ascertainment of similar and abnormal thyroid function tests in first degree relatives is suggestive (but certainly not diagnostic) of RTHβ. *THRB* sequencing is diagnostic in most patients and if a pathogenic variant is identified, genetic testing can be offered to first degree relatives with similar, abnormal TFTs. Increasingly, NGS identifies *THRB* variants of unknown significance; here, providing the variant *THRB* genotype cosegregates with abnormal thyroid function in families to establish pathogenicity, functional studies of *THRB* variants may not be necessary.

### 4.5 Treatment

Most individuals with RTHβ are asymptomatic not requiring specific treatment. Autonomic manifestations of hyperthyroidism (e.g., anxiety and palpitations) are responsive to beta‐blockade, with such therapy not affecting growth in childhood. A minority of patients experience more significant symptoms due to exposure of TRα‐expressing tissues to elevated circulating TH, including symptomatic tachycardia or persistent atrial fibrillation and impaired cardiac function, failure to thrive (infancy) and difficulty maintaining weight (adulthood) and anxiety or hyperactivity. In such cases, lowering TH levels may be helpful; options to achieve this include use of TRIAC (triiodothyroacetic acid, a TH analogue that preferentially acts centrally to inhibit TSH secretion, thereby lowering TH) or antithyroid drug (ATD) treatment.^59^ As therapy with ATDs results in a significant rise in TSH, driving goitre formation, potentially overcoming their inhibitory effect on TH synthesis and causing pituitary thyrotroph hyperplasia,^53^ our preference is to treat with TRIAC in the first instance, adding ATDs later if TRIAC alone is not sufficient to control symptoms. Total thyroidectomy or radioiodine treatment should be reserved as a last resort; following such thyroid ablation, thyroxine therapy in markedly supraphysiological dosage is required to normalise TSH levels, resulting in hyperthyroxinaemia of similar magnitude to before such interventions.

All patients with RTHβ should be followed long term, with suggested annual surveillance of adults including clinical assessment of symptoms, cardiovascular and thyroid examination and measurement of thyroid function and autoantibodies, fasting glucose and lipids. Cardiac telemetry may be warranted in cases with significant change in character or frequency of palpitations. Monitoring of bone health with periodic DXA scans and reviewing fracture history is also recommended. In children, autonomic and cardiac, thyrotoxic, symptoms, hyperactivity and educational performance, growth, goitre size and bone age should be monitored.

### 4.6 Resistance to thyroid Hormone α (RTHα)

Although α and β TH receptors are highly homologous, the equivalent human disorder (Resistance to thyroid Hormone α, RTHα), eludes diagnosis because it comprises many features of hypothyroidism in specific tissues, but associated with near‐normal thyroid function tests.

### 4.7 Molecular genetics

Twenty one different heterozygous pathogenic variants in *THRA*, mostly homologous to known variants of the equivalent amino acid in TRβ causing RTHβ and inherited from either parent or occurring ‘de novo’, have been documented^60^ (Figure 4B).

Many RTHα cases involve *THRA* variants which affect both TRα1 and TRα2 isoforms. When studied in the TRα2 protein background, these mutations exhibit no added gain or loss‐of‐function, which correlates with absence of any discernible additional clinical phenotype attributable to mutant TRα2, in these patients.^61^ Highly unusual clinical features (micrognathia, clavicular agenesis and syndactyly) associated with mutant TRα1 and α2 in a single patient, were not reproduced in a transgenic mouse model and may be unrelated to the *THRA* defect.^62^ Due to the absence of an overt thyroid biochemical phenotype, many *THRA* mutations are identified by NGS in childhood cases of delayed growth or neurodevelopment of unknown cause.

Similar to TRβ variants causing RTHβ, TRα1 mutants inhibit the function of their wild type receptor counterparts in a dominant negative manner.^63^

### 4.8 Clinical phenotype

Some features of CH (e.g., macroglossia, poor feeding and hoarse cry), have been recorded at birth. Abnormal physical characteristics include macrocephaly, dysmorphic facies with a flattened nose, prominent tongue and thick lips, together with an excess of skin tags and moles, especially in adults.^64^, ^65^

Growth retardation, affecting the lower segment disproportionately, resulting in childhood short stature, is a major mode of presentation. Radiological features include delayed fontanelle fusion and excessively serpiginous cranial sutures (‘wormian bone’ appearance), delayed dentition and bone age, with femoral epiphyseal dysgenesis in severe cases in childhood. Cranial and cortical hyperostosis in long bones, together with increased bone mineral density, is present in most cases, especially adults.

Neurocognitive features include delayed milestones (motor, speech) in childhood with impaired fine and gross motor coordination (dyspraxia) and variably reduced IQ.^66^ Many patients are on the autistic spectrum,^67^ with seizures recorded rarely in severe cases.

Reduced frequency of bowel movements is a common finding, with severe constipation being a significant problem in some cases. Bradycardia is typical, with metabolic rate (resting energy expenditure) being low in most patients. Transmission of TRα defects to offspring occurs from both males and females, suggesting that the disorder does not overtly compromise fertility.^64^

The most consistent pattern of thyroid function tests comprises low or low‐normal free T4, and high or high‐normal free T3, resulting in an abnormally low T4/T3 ratio; reverse T3 levels are subnormal in some, but not all, cases. A mild normocytic anaemia and raised muscle CK levels are consistent abnormalities.^60^

Overall, these observations are consonant with hormone resistance in organs (e.g., myocardium, skeletal muscle and gastrointestinal tract) expressing predominantly TRα1, with preservation of TH sensitivity in TRβ‐expressing tissues (hypothalamus, pituitary and liver).

### 4.9 Treatment

Thyroxine therapy of RTHα is beneficial, improving growth (total and lower segment height), increasing resting energy expenditure, thereby limiting weight gain, lowering elevated muscle CK levels and enhancing well‐being.^61^, ^68^, ^69^ Addition of growth hormone to thyroxine therapy in childhood does not result in further improvement in growth.^70^ In cases harbouring mutant TRα1 whose dysfunction is reversible at higher TH levels, treatment from early childhood might have ameliorated their phenotype^61^; even in adult life, thyroxine‐treated patients report improved constipation and self‐confidence.^69^ In virtually all cases thyroxine treatment does not improve anaemia.

Following thyroxine treatment in physiological dosage, TSH levels suppress readily with elevation of FT3 to supraphysiological levels, consonant with preserved TH sensitivity within the hypothalamic−pituitary−thyroid axis; serum SHBG rises slightly from high‐normal baseline levels; however, heart rate and cardiac parameters remain within the normal range.^68^

Whether long term thyroxine therapy is beneficial for growth and development or devoid of significant, adverse effects in TRβ‐expressing tissues, remains to be determined.
